# Deficiency in nucleoside diphosphate kinase B leads to endothelial activation of the hexosamine biosynthesis pathway and cardiac dysfunction

**DOI:** 10.1186/s12933-025-02633-8

**Published:** 2025-02-21

**Authors:** Feng Shao, Johanna Wieland, Yixin Wang, Merve Keles, Zenghui Meng, Santosh Lomada, Miao Qin, Veronika Leiss, Abel Martin-Garrido, Manuela Fuhrmann, Yi Qiu, Felix A. Trogisch, Christiane Vettel, Joerg Heineke, Yuxi Feng

**Affiliations:** 1 Experimental Pharmacology Mannheim, European Center for Angioscience (ECAS), Medical Faculty Mannheim, Heidelberg University, Ludolf-Krehl-Str. 13-17, 68167 Mannheim, Germany; 2 Department of Cardiovascular Physiology, European Center for Angioscience (ECAS), Medical Faculty Mannheim, Heidelberg University, 68167 Mannheim, Germany; 3 DZHK (German Center of Cardiovascular Research), Partner Site Heidelberg/Mannheim, Mannheim, Germany; 4 First Department of Medicine, Faculty of Medicine, University Medical Centre Mannheim (UMM), University of Heidelberg, 68167 Mannheim, Germany; 5 Department of Pharmacology, Experimental Therapy and Toxicology, University of Tübingen, 72074 Tübingen, Germany

**Keywords:** NDPKB, Endothelial cells, Cardiomyopathy, Diabetic complications, Hexosamine biosynthesis pathway, O-GlcNAc, Cardiac dysfunction, Extracellular matrix, hiPSC-CM, Pre-diabetic

## Abstract

**Background:**

Nucleoside diphosphate kinase B (NDPKB) deficiency in endothelial cells (ECs) promotes the activation of the hexosamine biosynthesis pathway (HBP), leading to vascular damage in the retina. The aim of this study was to investigate the consequences of NDPKB deficiency in the mouse heart.

**Methods:**

NDPKB deficient mice were used in the study. Echocardiography was employed to assess cardiac function in vivo. Characterization of contractility in hiPSC-derived cardiomyocytes (hiPSC-CMs) was measured with the IonOptix contractility system. Immunoblotting and immunofluorescence were carried out to analyze the expression and localization of proteins in cultured cells and left ventricles (LVs).

**Results:**

NDPKB deficient mice displayed impaired glucose tolerance and increased heart weight compared to controls. Echocardiographic analysis revealed an increase in the diastolic diameter of the left ventricular posterior wall (LVPW), a decrease in the early diastolic mitral valve E and E′ wave, and in the ratios of E/A and E′/A′ in NDPKB deficient hearts, suggesting cardiac hypertrophy and diastolic dysfunction. In line with cardiac dysfunction, the phosphorylation of myocardial phospholamban (PLN) and the expression of sarcoplasmic/endoplasmic reticulum Ca^2+^-ATPase 2 (SERCA2) in the NDPKB deficient LVs were significantly reduced. Moreover, the accumulation of collagen, fibronectin as well as the upregulation of transforming growth factor β (TGF-β), were detected in NDPKB deficient LVs. In addition, activation of the HBP and its downstream O-GlcNAc cycle was observed in the LVs and cardiac ECs (CECs) isolated from the NDPKB^−/−^ mice. Furthermore, a bipolar O-GlcNAc regulation was identified in CMs. O-GlcNAc was decreased in NDPKB-depleted CMs, while conditioned medium from NDPKB-depleted ECs significantly increased O-GlcNAc levels, along with contractile and relaxation dysfunction of the hiPSC-CMs, which was attenuated by inhibiting endothelial HBP activation.

**Conclusions:**

Deficiency in NDPKB leads to endothelial activation of the HBP and cardiac dysfunction. Our findings may highlight the crucial role of proper endothelial HBP in maintaining cardiovascular homeostasis.

**Supplementary Information:**

The online version contains supplementary material available at 10.1186/s12933-025-02633-8.

## Introduction

Diabetic cardiomyopathy (DC), one of the most common diabetic complications, is characterized by metabolic disturbances, impaired diastolic function, cardiac hypertrophy, and accumulation of extracellular matrix (ECM) [1, 2]. As a contractile unit of the heart, fully operative cardiomyocytes (CMs) are pivotal for the left ventricle’s (LV) function. Defects in insulin signaling under hyperglycemia decrease calcium sensitization and reduce sarcoplasmic calcium uptake in CMs, thus contributing to abnormal calcium flux during early DC pathogenesis [3, 4]. In the pathology of DC, vascular cells, particularly endothelial cells (ECs), undergo significant alterations that contribute to vascular damage. In streptozotocin (STZ)-induced diabetic hearts, EC apoptosis increases, accompanied by decreased capillary density in LVs and diminished vascular endothelial growth factor (VEGF) expression [5]. ECs along with cardiac fibroblasts (CFs) are essential contributors to excessive deposition of ECM proteins, which has been reported not only in DC but as well in the pre-diabetic conditions [6, 7].

Abnormalities in glucose metabolism correlate with the initiation and development of DC. Increased flux of glucose through the polyol pathway, elevated intracellular formation of advanced glycation end products, activation of protein kinase C, and raised flux through the hexosamine biosynthesis pathway (HBP) are key mechanisms contributing to hyperglycemia-induced cellular damage [8, 9]. The HBP is an alternative fate of glucose that under physiological conditions diverts only a small amount of fructose-6-phosphate away from glycolysis. The conversion of fructose 6-phosphate to glucosamine 6-phosphate, representing the rate-limiting step in this process, is catalyzed by glucose-6-phosphate-Glutamine: fructose-6-phosphate amidotransferase (GFAT) [10]. Subsequently, UDP-GlcNAc, the end product of the HBP, is required as a substrate for protein O-GlcNAcylation, a post-translational modification involving the attachment of O-linked beta-N-acetylglucosamine (O-GlcNAc) to proteins by O-GlcNAc transferase (OGT), which can be reversed by the removal of O-GlcNAc through O-GlcNAcase (OGA) [11].

Studies have demonstrated that hyperglycemia provokes abnormal protein O-GlcNAcylation, implicating it in insulin resistance, mitochondrial dysfunction, oxidative stress, inflammatory activation and disturbances in CM calcium handling, thereby contributing to DC development [12–15]. Total protein O-GlcNAcylation levels increase in the myocardium of diabetic patients and correlate with LV dysfunction [16]. Park et al. have reported that O-GlcNAc is elevated in erythrocytes of pre-diabetic patients [17]. Nevertheless, it is unclear whether O-GlcNAc is altered in the pre-diabetic heart. Additionally, excessive cardiac O-GlcNAc levels have been observed in diabetic animal models of both type 1 and type 2 diabetes [18–20]. Enhanced O-GlcNAcylation, in conjunction with decreased protein phosphorylation, particularly of phospholamban (PLN), disrupts CM calcium flux, impacting cardiac diastolic function [21]. Overexpression of OGA has been shown to reduce protein O-GlcNAcylation and improve CM calcium handling and contractile function in diabetic hearts [22].

Nucleoside diphosphate kinases (NDPKs) are housekeeping enzymes involved in nucleotide homeostasis and various cellular processes, such as cell proliferation, differentiation, adhesion, and apoptosis [23–25]. Recently, studies indicate that NDPKB is involved in vascular homeostasis, especially in ECs. Enhanced protein O-GlcNAcylation observed in NDPKB deficient ECs contributes to vascular damage, leading to the development of pathological changes and diabetes-related complications such as retinopathy in NDPKB deficient mice [26, 27]. Given the pivotal role of ECs in organ function, we hypothesized that endothelial HBP may play a role in maintaining cardiac homeostasis. Therefore, we investigate the consequences of NDPKB deficiency in the mouse heart.

## Methods

### Animal model

Animal care and experiments were performed in accordance with the “2010/63/EU” statement of the European Parliament and were approved by Regierungspräsidium Karlsruhe (reference number: G-165/20) and the Medical Faculty Mannheim, Heidelberg University (reference number I-22/09). Genotypes of NDPKB deficient (NDPKB^−/−^) male mice with a C57BL/6 background were determined as described previously [28]. Age-matched wild-type (WT) mice served as controls. Blood glucose was measured using a glucometer (Sanofi, Germany). At the age of 14 months, echocardiography was performed to determine ventricular function, heart size, and mass. Subsequently, mice were sacrificed by isoflurane followed by cervical dislocation, and hearts were removed for further analysis. General parameters such as blood glucose, glycated hemoglobin (HbA1c) (Infopia Clover A1c Analyzer, EuroMedix, Germany), heart weight, body weight and tibia length were measured. Hearts were dissected, residual blood was washed out with 0.9% NaCl solution, and the middle vertical parts were cut out and fixed in 4% formalin for paraffin sections or embedded in Tissue Tek O.C.T (4583, Sakura Finetek, USA) for cryosections. The LVs were isolated from the rest of the hearts, shock-frozen in liquid nitrogen, and stored at − 80 °C until further use.

### GTT and ITT

After a 4-h fasting period, blood samples were taken from the tail of the 8-month-old mice to measure fasting blood glucose levels using a glucometer (Roche, Germany). Mice were subsequently administered glucose intraperitoneally at a dosage of 1 g/kg of body weight for the glucose tolerance assessment (GTT), or subcutaneous injection of insulin at a rate of 1 U/kg of body weight (Lantus insulin glargine Sanofi, Germany) for insulin tolerance test (ITT). Blood glucose levels were measured at 15-, 30-, 60-, 90- and 120-min post-injection for GTT, and at 30-, 60-, 90- and 120-min post-injection for ITT.

### Isolation of pancreatic islets and measurement of insulin and glucagon release

Pancreatic islets were isolated from 10–12-month NDPKB^−/−^ and control mice using a protocol described previously [29]. Briefly, after cervical dislocation, 1.9 U/mL collagenase type 4 (Worthington, LS004186, Nagold, Germany) was injected into the pancreas via the bile duct. The pancreas was removed, digested at 37 °C for 10 min, and islets were isolated and transferred to culture medium (RPMI 1640 medium (ThermoFisher, 31870025, Karlsruhe, Germany) supplemented with 1% penicillin–streptomycin (Sigma-Aldrich, P4333, Taufkirchen, Germany) and 10% FBS (Sigma-Aldrich, S0615, Taufkirchen, Germany)). The insulin secretion was assessed in cultured islets treated with high glucose (Sigma Aldrich, G7021, Taufkirchen, Germany) (16 mM) compared with controls (3 mM glucose) [30]. Insulin levels in the supernatant were measured using an ultra-sensitive insulin ELISA kit (DRG Diagnostics, Marburg, Germany). For glucagon secretion, islets incubated in Krebs-HEPES buffer were exposed to 1 mM or 10 mM glucose for 1 h. Glucagon levels in the supernatant were measured by a radioimmunoassay kit (Linco Research, Inc, St. Charles, MO, USA).

### Transthoracic echocardiography

Echocardiography was performed under standard protocols with isoflurane for anesthesia (maintenance at 1% after induction with 3% isoflurane with 1 Lpm oxygen). Since diastolic dysfunction precedes systolic dysfunction in DC, and an anesthesia-mediated decrease in heart rate is required for proper assessment of diastolic dysfunction because of the high physiological beating frequency in small rodents, we recorded the echocardiography in anesthetized mice. Briefly, mice were placed in a dorsal position on an examination table with an adjusted heater to maintain the body temperature at 37 °C under continuous electrocardiogram and respiration rate recordings. Echocardiography was recorded with the Vevo 3100 system (VisualSonics, Toronto, Canada) using a linear 30–40 MHz transducer (MX-550D). Data were analyzed with the Vevo Lab 5.5.0 software [31].

For assessment of LV structure and function, B-mode and M-mode traces were recorded at the papillary muscle level. LV posterior wall thickness (LVPW) and end-diastolic volume measurements were used to characterize LV anatomy. The change in LV diameter in length from end-diastole to end-systole was used to assess contractility and to calculate LV ejection fraction and fractional shortening. B-mode tracings in the apical four-chamber view were recorded at the atrioventricular valve level with pulsed-wave Doppler (PW-Doppler) and tissue-Doppler measurements including mitral valve (MV) E, A, E′ and A′.

To measure the functional and anatomical features of the LV, auto LV analysis was performed using the Vevo Lab echo analysis software. To measure the systolic parameters, the left ventricular trace was measured in M-mode and the endo- and epicardium were selected through multiple cardiac cycles. For the measurement of the diastolic function, the color Doppler mode was optimized for mitral valve flow. To assess the mitral valve flow in PW Doppler mode, the respective A traces or E traces were measured. Additionally, the mitral valve annulus movement parameters, such as MV A′ or MV E′, in tissue-Doppler mode were calculated as well.

### Protein extraction and immunoblotting

The LV was frozen in liquid nitrogen, ground into powder and transferred into RIPA lysis buffer (50 mM Tris–HCl, pH7.4, 150 mM NaCl, 1 mM dithiothreitol, 1% Triton X-100, 1% sodium deoxycholate, protease inhibitor (cOmplete™ Protease Inhibitor Cocktail, 11836145001, Roche, Germany), and phosphatase inhibitor (PhosSTOP™ phosphatase inhibitor tablets, 4906837001, Roche, Germany), and homogenized by a TissueLyser II (300 Hz; 4 × 30 s, Qiagen, Germany) with the stainless steel 7 mm beads (69990, Qiagen, Germany). Following centrifugation, the supernatants containing the extracted proteins were collected and mixed with Laemmli buffer. The proteins were then denatured at 95 °C for 5 min, separated by SDS-PAGE, and subsequently transferred onto a nitrocellulose membrane via wet electroblotting in a transfer tank. After blocking with Roti-block (A151.2, Roth, Karlsruhe, Germany) for 1 h at room temperature, the transferred proteins were probed with the primary antibodies at 4 °C overnight. After washing the next day, membranes were incubated with corresponding secondary antibodies for 1 h at room temperature. Immune complexes were visualized using chemiluminescent peroxidase substrates (Lumi light, 2015200, Roche, Mannheim, Germany; or Femto, 34095, Thermo Scientific, Rockford, USA). Protein levels were analyzed using ImageJ software (NIH, USA). The primary antibodies used in this study were: mouse-anti-NDPKB (1:2000, Acris MC412); mouse-anti-PLN (dilution 1:5000, Badrilla A010-14); rabbit-anti-PLN pSER16 (dilution 1:5000, Badrilla A010-12); rabbit-anti-SERCA2 (dilution 1:1000, Santa Cruz sc-8094); mouse-anti-O-GlcNAc (dilution 1:1000, Abcam ab2739); rabbit-anti-GFAT (dilution 1:3000, a gift of Prof. E. Schleicher, Tübingen); rabbit-anti-OGT (dilution 1:1000, Sigma Aldrich O6264); rabbit-anti-OGA (dilution 1:1000, Sigma Aldrich SAB4200267); goat-anti-VE-cadherin (dilution 1:1000, Santa Cruz sc32322); rabbit-anti-Fibronectin (dilution 1:1000, Sigma F3648); mouse-anti-TGF-beta1 (dilution 1:500, Santa Cruz sc52893); mouse-anti-CTGF (dilution 1:500, Santa Cruz sc365970); mouse-anti-γTubulin (dilution 1:3000, Sigma T6557). Corresponding secondary antibodies used were goat-anti-mouse IgG (H + L) (dilution 1:10000, Invitrogen G21040); rabbit-anti-goat IgG (dilution 1: 10000, Sigma A8919); goat-anti-rabbit IgG (H + L) (dilution 1:10000, Invitrogen G21234).

### Immunofluorescence staining

Paraffin sections (5 μm) were heated at 60 °C for approximately 1 h until the paraffin ran down. After cooling, the slides were deparaffinized by xylene and rehydrated by serial dilutions of ethanol. Antigen retrieval was carried out by microwave heating for 15 min in a citrate antigen retrieval solution containing citric acid monohydrate and trisodium citrate dihydrate (pH 6) followed by blocking. Cryosections were fixed in cold acetone for 10 min and then air-dried at room temperature (RT) for 10 min. Blocking and permeabilization were achieved by incubating the samples in a solution with 1% BSA and 0.5% Triton X in PBS at RT for 10 min. Afterwards, the sections were incubated with the primary antibodies at 4 °C overnight. The next day, after washing, samples were probed with the corresponding secondary antibodies in a dark chamber for 1 h at RT. Finally, samples were covered with mounting media (ROTI® Mount FluorCare, HP19.1, CARL ROTH, Germany) and all the samples were visualized under a confocal microscope (Leica TCS sp8, Wetzler, Germany). Non-overlapping images from the posterior wall of LVs were obtained for quantification. Primary antibodies included were rabbit-anti-Fibronectin (dilution 1:200, Sigma Aldrich F3648); mouse-anti-O-GlcNAc (dilution 1:200, Abcam ab2739); Lectin-TRITC (dilution 1:50, Sigma Aldrich L5264); Lectin-FITC (dilution 1:100, Sigma Aldrich L9381); rabbit-anti-CD31 (dilution 1:200, Santa Cruz sc1506R). Corresponding secondary antibodies used were swine-anti-rabbit FITC (dilution 1:20, Dako, F0205); goat-anit-mouse 488 (dilution 1:200, Invitrogen A21121); swine-anti-rabbit TRITC (dilution 1:20, Dako, R0156); goat-anti-rabbit cy3 (dilution 1:200, Jackson Laboratories 111-165-144). For determination of cardiac myocyte size staining with wheat germ agglutinin (WGA) was used. Six images were obtained from the subendocardial and subepicardial layer of the myocardium of the posterior wall in LVs. ImageJ was used to quantify the size of CMs [32]. To evaluate capillary density, vessels were stained with Lectin-FITC. Images were captured, and the capillary density was determined by calculating the ratio of the lectin-stained areas to the total tissue areas.

### Picro sirius-red staining

Picro Sirius-red Staining was performed according to the manufacturer’s protocol (Niepötter Labortechnik, Bürstadt, Germany). Paraffin sections were deparaffinized, hydrated and subsequently stained in Picro Sirius Red Solution for 60 min at room temperature. After dehydration, slides were mounted in Entellan (Sigma-Aldrich, Taufkirchen, Germany). Accumulation of collagens was evaluated using ImageJ in whole heart section or in subendocardial and subepicardial layer of myocardium in LVs for each heart section.

### Isolation and culture of CFs from NDPKB^−/−^ mice

Ventricles from NDPKB^−/−^ mice were minced into small pieces of 1–2 mm and then digested with corresponding digestion buffer containing 0.12 U/mL dispase (Corning, 354235, Wiesbaden, Germany), 0.012% trypsin (ThermoFisher Scientific, 15090046, Karlsruhe, Germany), 1.6 mg/mL collagenase type 1 (Worthington, LS004197, Nagold, Germany) and 1.33 U/mL DNase type 1 (Merck, DN25, Darmstadt, Germany). The supernatant was collected, and the reaction was stopped by adding culture Dulbecco's Modified Eagle Medium (DMEM) (Sigma-Aldrich, D5546, Taufkirchen, Germany) containing 10% FBS and 1% Penicillin–Streptomycin. After repeating the digestion eight times, cells were centrifuged, re-suspended and then seeded into the plate. Unattached cells and debris were eliminated by gentle washing with culture medium after 2 h. Cells at passage 2 were seeded into a 6-well plate and used for harvesting proteins, followed by immunoblotting.

### Isolation of cardiac ECs (CECs) from NDPKB^−/−^ mice

CECs were extracted using the MACS-magnetic CD146-microBeads (Miltenyi Biotec, 130-092-007, Germany) [33]. Briefly, mouse cardiac ventricles were minced and placed into RPMI 1640 medium containing 500 U/mL collagenase type 1 and 150 U/mL DNase I (Worthington, LS002139, Nagold, Germany). Following an incubation at 37 °C for 45 min, digestion mixture was passed through a 70 μm cell strainer. CECs were then combined with CD146 microbeads in MACS buffer (Miltenyi Biotec, 130-091-222, Radolfzell, Germany) for an additional 30 min using a magnetic column (Miltenyi Biotec, 130-042-201, Radolfzell, Germany). Proteins were extracted from CECs directly after elution and subjected to immunoblotting.

### HUVECs culture

The utilization of human umbilical vein endothelial cells (HUVECs) was approved by the local medical ethics committee (Medical Faculty Mannheim, Heidelberg University, Germany). Under the mother’s consent, the umbilical cords of healthy newborn babies were used for HUVECs isolation. Clamps were disinfected by baking at 180 °C for 4 h. HUVECs were digested with 1 mg/mL dispase (Sigma-Aldrich, 4,942,078,001, Taufkirchen, Germany) and neutralized with 10% FBS. Cells were suspended in the endothelial basal medium containing 2% FBS (ECBM, PromoCell, C-22210, Heidelberg, Germany) and seeded in 1% gelatin-coated culture flasks with 2% FBS in a humidity incubator at 37 °C and 5% CO_2_. The culture medium was changed after 2 h to remove excess red blood cells. Cells were passaged until confluence in ECBM with 10% FBS (completed ECBM) and used from passage 2 to 3 for experiments.

### siRNA transfection

HUVECs were cultured in 1% gelatin-coated 6-well plates with completed ECBM at a humidity incubator at 37 °C and 5% CO_2_ overnight until they reached 70–80% confluence. Cells were incubated in OptiPRO SFM (serum-free medium) (ThermoFisher Scientific, 2026928, Karlsruhe, Germany) and lipofectamine RNAiMAX (Invitrogen, 2463615, Karlsruhe, Germany) together with NDPKB-specific siRNA 5′-AGG UAG UGU AAU CGC CUU G-3′ (Eurofins, MWG, Germany) or scrambled siRNA 5′-AAC UGG UUG ACU ACA AGU CUU-3′ (Eurofins MWG, Germany) and OGT-specific siRNA SI02631412 (Qiagen, Germany) or Scramble siRNA SI03650318 (Qiagen, Germany) according to the manufacturer’s protocol for 4 h. Cells were subsequently cultured overnight in completed ECBM followed by starvation with ECBM containing 0.5% FBS for 48 h, after which cellular proteins were harvested for immunoblotting and media were collected for human induced pluripotent stem cell-derived cardiomyocyte (hiPSC-CM) experiment.

For transfection of hiPSC-CMs, hiPSC-CMs were cultured in a 24-well plate and incubated overnight in OptiPRO SFM, along with Lipofectamine RNAiMAX and NDPKB-specific siRNA or Scramble siRNA. The following morning, cells were transferred to complete medium. After 48 h, videos were recorded using an Olympus cellSens Microscope, and cellular proteins were harvested for immunoblot analysis.

### Culture of hiPSC-CMs treated with conditioned medium and contractility measurement

hiPSC-CMs were differentiated from healthy donor-derived human induced pluripotent stem cells as previously described [34]. After 50 days of differentiation, the hiPSC-CMs were dissociated into single cells and seeded onto a 24-well culture plate. Subsequently, the hiPSC-CMs were exposed to control, NDPKB depleted, OGT depleted or NDPKB/OGT double depleted HUVECs culture medium for 12 h. The cells were harvested for immunoblotting or contractility measurement. For contractility measurement, the medium was replaced with physiological saline solution (PSS) containing: 127 NaCl mM, 5.9 KCl mM, 2.4 CaCl₂ mM, 1.2 MgCl₂ mM, 11 mM glucose, 10 mM HEPES, pH 7.4. The contractility of spontaneously beating hiPSC-CMs was recorded using the IonOptix contractility system (IonOptix, USA), which is equipped with the high-speed MyoCam-S contractility camera and the SoftEdge™ Acquisition module. The measurements were analyzed using the IonWizard 6.6.9 system (IonOptix, USA), and the following key parameters of contractility were reported: beat frequency (Hz), time to % peak (contraction kinetics, duration from baseline to % peak), and time to % baseline (relaxation kinetics, duration from peak return to % baseline). Each beating hiPSC-CM was continuously measured for 60 s, and approximately 15 beating hiPSC-CMs were evaluated per well. This procedure was repeated in three independent experiments for each of the two culture conditions.

### Statistical analysis

The data are presented as mean ± SD. The results were analyzed by unpaired t-test or ANOVA following a normal distribution, otherwise by Kruskal–Wallis-test. GraphPad Prism 5 (GraphPad Software, La Jolla, CA, USA) was utilized for the statistical analysis. p values < 0.05 were considered significant.

## Results

### NDPKB deficient mice show increased heart weight and cardiac hypertrophy

**Fig. 1:**
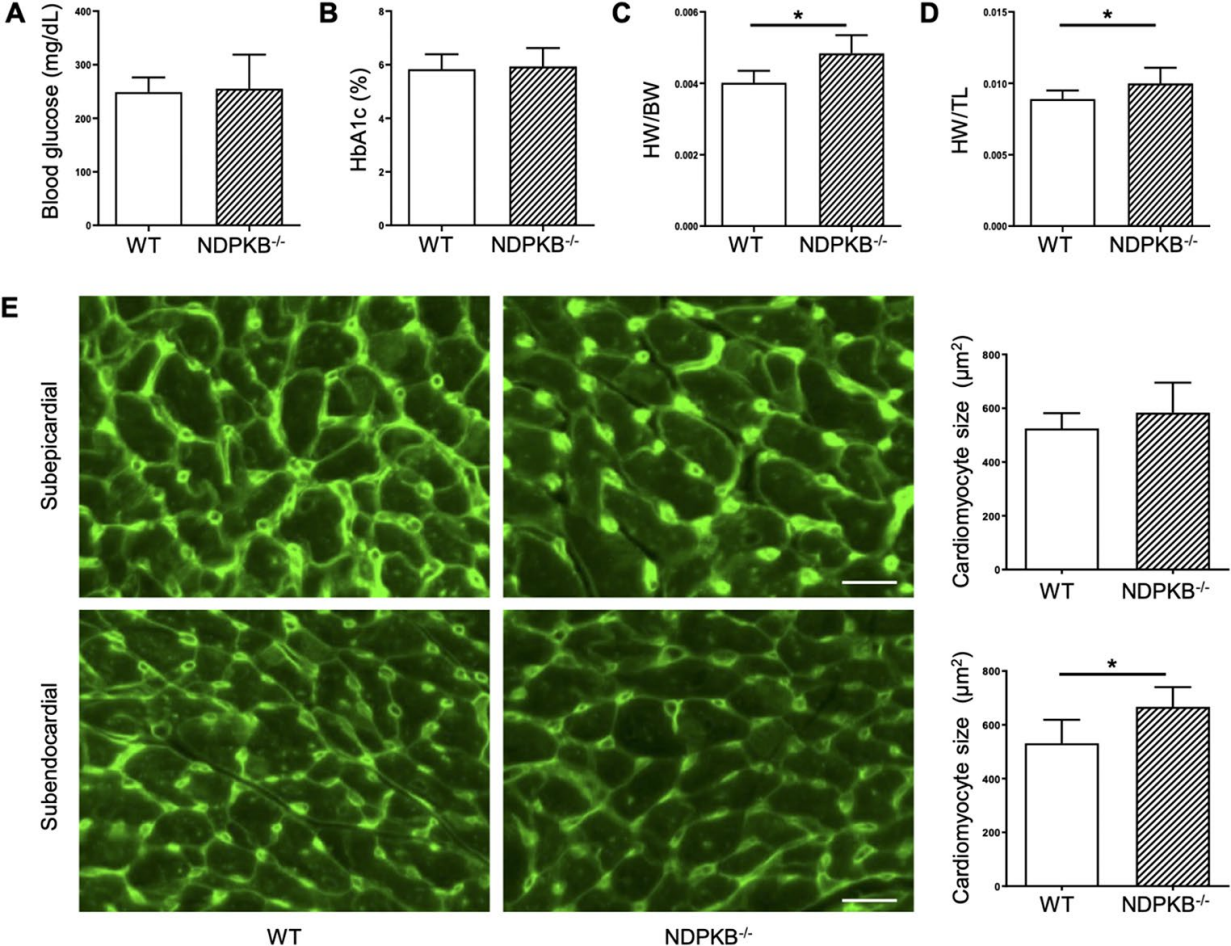
NDPKB deficient mice show cardiac hypertrophy. General parameters are shown: Blood glucose levels (**A**), HbA1c (**B**), the ratio of heart weight to body weight (**C**), and the ratio of heart weight to tibia length (**D**). n = 10. Representative images and statistical analysis of CM size via WGA staining from subepicardial and subendocardial layers in NDPKB^−/−^ heart sections (**E**). n = 5. *p<0.05. Scale bar = 50 µm

To investigate the consequences of NDPKB deficiency in the mouse heart, general parameters in NDPKB^−/−^ mice were assessed. There was no difference in body weight between NDPKB^−/−^ and WT control mice (WT: 39.85 ± 0.80 g, NDPKB^−/−^: 36.75 ± 1.55 g, p > 0.05). Blood glucose levels in NDPKB^−/−^ mice were comparable to those in their control mice as well (p > 0.05, Fig. 1A). HbA1c levels, a gold standard for monitoring long-term alteration of blood glucose, were similar in both groups (p > 0.05, Fig. 1B). To better characterize the metabolic status of NDPKB^−/−^ mice, we additionally performed the GTT and ITT. An increased trend in blood glucose was observed in the NDPKB^−/−^ mice after 4 h of fasting, but did not reach significance. Regarding GTT, NDPKB^−/−^ mice showed significant elevation of blood glucose levels compared to controls at 15 and 30 min (Supplementary Fig. 1A). However, blood insulin levels at 0 and 30 min during GTT were unchanged in NDPKB^−/−^ compared to WT mice (Supplementary Fig. 1B). Subsequently, the ITT was carried out to estimate insulin sensitivity in the mice. The ITT results showed no differences in blood glucose between NDPKB^−/−^ and WT mice (Supplementary Fig. 1C). Furthermore, pancreatic islet function was assessed in isolated islets from the NDPKB^−/−^ mice. As demonstrated in supplementary Fig. 1D and 1E, no significant differences were observed between NDPKB^−/−^ and WT mice in either insulin or glucagon secretion. The results indicate that NDPKB^−/−^ mice exhibit pre-diabetic characteristics such as impaired glucose tolerance with normal insulin secretion, and normal insulin sensitivity.

NDPKB^−/−^ mice exhibited no difference in heart weight compared to the control mice (WT: 0.16 ± 0.003 g, NDPKB^−/−^: 0.18 ± 0.007 g, p > 0.05). Unexpectedly, normalized heart weight to body weight in NDPKB^−/−^ mice was significantly increased (HW/BW, p < 0.001, Fig. 1C). To mitigate the effect of individual body weight, heart weight was also normalized to tibia length, which remains stable after maturation. The ratio of heart weight to tibia length was elevated in NDPKB^−/−^ mice (HW/TL, p < 0.01, Fig. 1D). Hagdorn et al. recommended standardization to TL to the power of three (TL^3^) as a correct surrogate for cardiac measurement in mice [35]. Standardization to TL^3^ also confirmed a significant gain in heart weight to tibia ratios, approximately by 13%, in NDPKB^−/−^ compared with WT mice (HW/TL^3^, WT: 6.87 ± 0.16 mg/mm^3^, NDPKB^−/−^: 7.75 ± 0.29 mg/mm^3^, p < 0.05). Lung weights showed no significant differences between the two groups (WT: 0.17 ± 0.01 g, NDPKB^−/−^: 0.17 ± 0.01 g, p > 0.05). We further investigated CM morphological alterations in the NDPKB deficiency in the LV posterior wall. Heart sections from NDPKB^−/−^ mice showed enlarged ventricular CMs in the subendocardial layer of the myocardium compared to WT counterparts. No difference was observed between the two groups in the subepicardial layer (Fig. 1E). The data indicate that NDPKB deficiency in the heart may induce cardiac hypertrophy in normo-glycemic pre-diabetic mice.

### NDPKB deficient mice exhibit left ventricular diastolic dysfunction

**Fig. 2:**
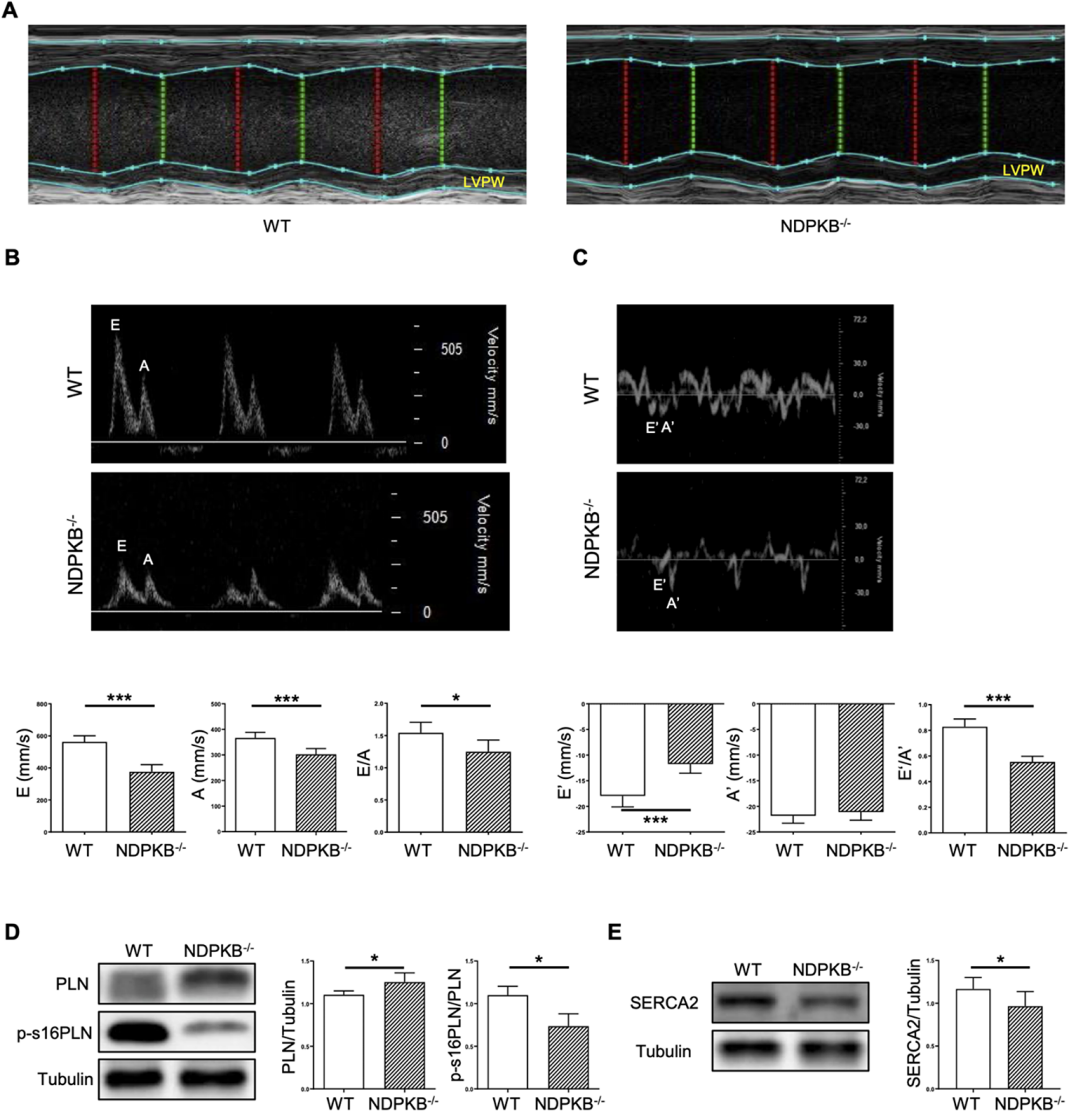
Echocardiography manifests diastolic dysfunction in NDPKB deficient mice. The thickness of the LVPW end diastole (**A**). Representative echocardiography traces of pulsed-wave flow Doppler imaging, E velocity, A velocity and the ratio of E to A (**B**). Representative echocardiography traces in A4C view of mitral valve tissue Doppler imaging, E’ velocity, A’ velocity and the ratio of E’ to A’ (**C**). Immunoblotting and statistical analysis of PLN and phospho- PLN in LVs (**D**). Immunoblotting and statistical analysis of SERCA2 in LVs (**E**). n = 6. *p < 0.05, ***p < 0.001

To determine the impact of NDPKB deficiency on cardiac function, echocardiography was performed in the mice. Measurements of the LVs were assessed using M-Mode echocardiography, whereas microvascular flow was visualized and evaluated using the PW-Doppler. NDPKB deficient hearts did not show significant changes in heart rate, systolic diameter, diastolic diameter, cardiac output, ejection fraction or fractional shortening compared to WT mice (Table 1). Lower absolute levels in ejection fraction are a consequence of anesthesia applied during the echocardiographic recording as reported by Berry et al. [36]. Additionally, there were no significant differences in stroke volume, systolic volume, diastolic volume, systolic LV anterior wall (LVAW; systole), diastolic LV anterior wall (LVAW; diastole), nor systolic LVPW (LVPW; systole) between the two groups (Table 1). In NDPKB^−/−^ hearts, a significant increase in LV mass and its correlation was observed, providing strong evidence that the primary enlargement occurred within the LV. This was further supported by the finding that the diastolic thickness of the LVPW (LVPW; diastole) was significantly greater in NDPKB^−/−^ hearts compared to the control group (Table 1, Fig. 2A, p < 0.05). These results collectively highlight the hypertrophic response predominantly localized to the LVs in NDPKB^−/−^ hearts. Moreover, PW-doppler examinations showed that peak ventricular filling velocity in early diastole (passive flow) (E) (Fig. 2B, p < 0.05) was significantly reduced, meanwhile the peak velocity in late diastole (atrial contraction) (A) (Fig. 2B, p < 0.05) was also altered in NDPKB^−/−^ hearts compared to the control hearts. It is worth noting that the MV E-wave/A-wave ratio significantly decreased (Fig. 2B, p < 0.05), suggesting inefficient LV filling, and referring to the altered diastolic performance of the NDPKB^−/−^ hearts. Moreover, levels of the early mitral annular velocity (E′) (Fig. 2C, p < 0.01) were significantly lower in NDPKB^−/−^ mice than in controls, and the late mitral annular velocity (A′) (Fig. 2C, p > 0.05) was similar in both groups. The ratio of E′ to A′ was significantly decreased (Fig. 2C, p < 0.05). However, the ratio of E to E′ was unchanged in both NDPKB^−/−^ and WT groups (WT: − 31.38 ± 1.65, NDPKB^−/−^: − 32.51 ± 2.27, p > 0.05). The data points out that the NDPKB deficient mice exhibit reduced passive filling in the LVs properly due to impaired ventricular relaxation. The echocardiography results indicate that the diastolic dysfunction in the LVs of NDPKB deficient mice may be caused by insufficient filling during the early diastole.

**Table 1:** Echocardiography of NDPKB deficient mice shows LV hypertrophy. n = 6

Parameter	WT	NDPKB ^−/−^	p value
Heart Rate (bpm)	471.20 ± 21.42	452.40 ± 49.11	p = 0.41
LV Diameter; systole (mm)	3.49 ± 0.27	3.52 ± 0.22	p = 0.85
LV Diameter; diastole (mm)	4.27 ± 0.21	4.23 ± 0.18	p = 0.74
LV Volume; systole (μl)	50.85 ± 9.34	51.65 ± 7.94	p = 0.88
LV Volume; diastole (μl)	81.93 ± 9.53	80.18 ± 8.16	p = 0.74
Stroke Volume (μl)	31.08 ± 4.54	28.53 ± 3.64	p = 0.31
Ejection Fraction (%)	38.23 ± 6.03	35.75 ± 4.64	p = 0.44
Fractional Shortening (%)	18.36 ± 3.21	16.97 ± 2.49	p = 0.42
Cardiac Output (mL/min)	14.66 ± 2.28	12.96 ± 2.57	p = 0.25
LV Mass (mg)	140.20 ± 5.73	173.3 ± 28.75	*p = 0.02
LV Mass Cor (mg)	112.10 ± 4.58	138.60 ± 23.00	*p = 0.02
LVAW; systole (mm)	1.22 ± 0.16	1.31 ± 0.20	p = 0.42
LVAW; diastole (mm)	0.91 ± 0.08	0.99 ± 0.11	p = 0.18
LVPW; systole (mm)	0.98 ± 0.09	1.12 ± 0.16	p = 0.08
LVPW; diastole (mm)	0.77 ± 0.06	0.99 ± 0.18	*p = 0.02

To gain insights into the cardiac dysfunction, we further estimated the phosphorylation of PLN and sarcoplasmic/endoplasmic reticulum Ca^2+^ -ATPase 2 (SERCA2), which together modulate Ca^2+^ dynamics and thus regulate CM contractility and relaxation, in the LVs by immunoblotting. Notably, we found that the expression of total PLN was increased, while phosphorylation of PLN at the residue S16 was significantly declined in NDPKB^−/−^ LVs. The diastolic dysfunction observed in the NDPKB deficient mice may be based on altered SERCA2 activity that is closely associated with PLN regulation. In addition, downregulation of SERCA2 has been reported for different etiologies, including diabetes. To investigate this further, we performed immunoblotting and observed a significant decrease about 25% in SERCA2 expression in the NDPKB^−/−^ LVs (Fig. 2D and E). This decrease in PLN-S16 phosphorylation and SERCA2 downregulation imply impaired calcium cycling and reduced efficiency of myocardial contraction/relaxation velocities. These data suggest that NDPKB deficiency causes diastolic dysfunction likely via interfering with the key modulators of the CM contraction and relaxation cycle.

### NDPKB deficiency prompts the accumulation of ECM in LVs

**Fig. 3:**
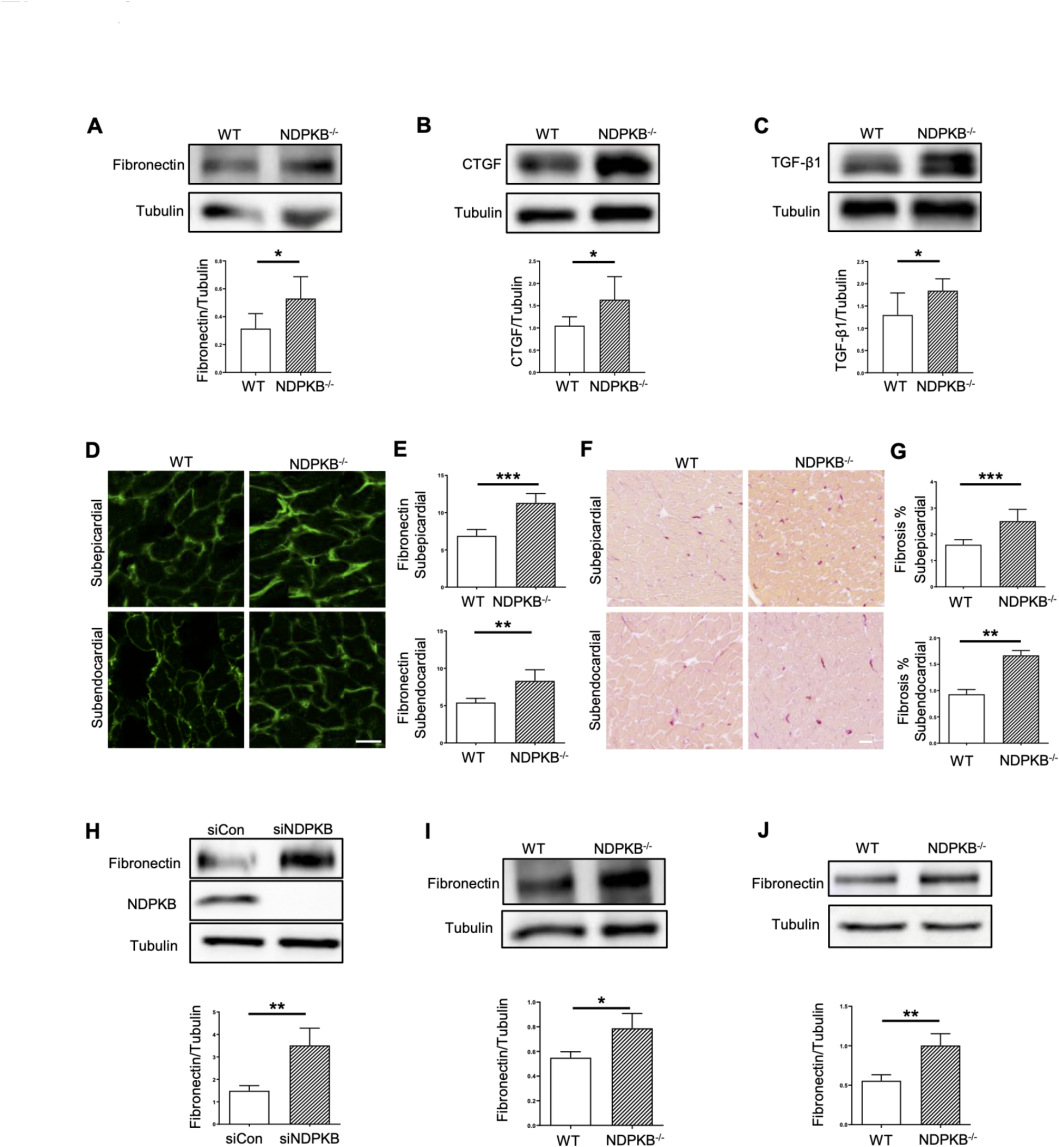
Accumulation of ECM is evident in the LVs of NDPKB deficient mice. Immunoblotting and statistical analysis of fibronectin (**A**), CTGF (**B**) and TGF- β1 (**C**) in NDPKB^−/−^ LVs. n = 6. Immunofluorescence staining (**D**) and the corresponding statistical analysis of fibronectin in the subepicardial and subendocardial layers (**E**). n = 5, repeated for three times. Scale bar = 50 µm. Sirius-red staining (**F**) and statistical analysis of collagen in the subepicardial and subendocardial layers (**G**). n = 5, repeated for three times. Scale bar = 20 µm. Immunoblotting and statistical analysis of fibronectin in NDPKB-depleted HUVECs (**H**). n = 4. Immunoblotting and statistical analysis of fibronectin in NDPKB^−/−^ and WT CECs (**I**). n = 4. Immunoblotting and statistical analysis of fibronectin in NDPKB^−/−^ and WT CFs (**J**). n = 4. *p < 0.05, **p < 0.01, ***p < 0.001

Diastolic dysfunction is closely associated with the mechanical properties of ventricular tissue and thus enhanced ECM production as seen under pathological conditions such as DC with the primarily source being ECs and fibroblasts. We further analyzed the expression of ECM proteins using immunoblotting. The expressions of fibronectin and CTGF (Fig. 3A and B) were significantly increased. TGF-β could induce the deposition of the ECM components, contributing to the development of cardiac fibrosis. Expectedly, significantly elevated expression of TGF-β1 was detected in NDPKB^−/−^ LVs (Fig. 3C), validating previous data on the accumulation of ECM proteins in LVs. Subsequently, we estimated the localization of accumulated ECM proteins using immunofluorescence staining of cryosections. Given our prior findings of cardiac hypertrophy in the LVPW of NDPKB^−/−^mice we first focused on the expression of fibronectin in these regions. We analyzed the subendocardial and subepicardial layer of myocardium in LVs and found that in NDPKB^−/−^ LVs, fibronectin was mostly expressed in vascular and peri-vascular regions (Supplementary Fig. 2). Notably, expression of fibronectin was higher in the subepicardial layer than in the subendocardial layer (Fig. 3D), and it was upregulated in both subendocardial and subepicardial layers in NDPKB^−/−^ mice compared to controls (Fig. 3E). To validate our findings, we performed Sirius-red staining, which specifically binds to collagen fibers, and measured collagen stained red in paraffin sections. The collagen accumulation was increased across the entire heart sections (Supplementary Fig. 3). A 44% and 88% increase in collagen deposition was observed in the subepicardial and subendocardial layer in NDPKB^−/−^ LVs respectively (Fig. 3F and G). These data underscore that the NDPKB^−/−^ mice display ECM accumulation in the LVs. Given the fact that loss of NDPKB has a significant effect on EC function [26, 27, 37], we investigated the impact of NDPKB depletion on ECM production in HUVECs. The immunoblotting experiments showed that fibronectin levels were significantly increased in NDPKB-depleted ECs (Fig. 3H). To confirm the findings in HUVECs, we isolated CECs from the NDPKB^−/−^ and WT mice. Our immunoblotting data showed a 50% upregulation of fibronectin expression in CECs of NDPKB^−/−^ mice, confirming the data observed in HUVECs (Fig. 3I). Based on the essential role of CFs in fibrosis in the heart, expression of fibronectin was also assessed in CFs isolated from the NDPKB^−/−^ and WT hearts. An approximate twofold increase in fibronectin expression was detected in CFs isolated from NDPKB^−/−^ mice (Fig. 3J). Collectively, these data demonstrated that NDPKB deficiency results in cardiac ECM accumulation which could be primarily driven by CECs and CFs.

### The hexosamine biosynthesis and downstream pathways are activated in NDPKB deficient hearts and CECs

**Fig. 4:**
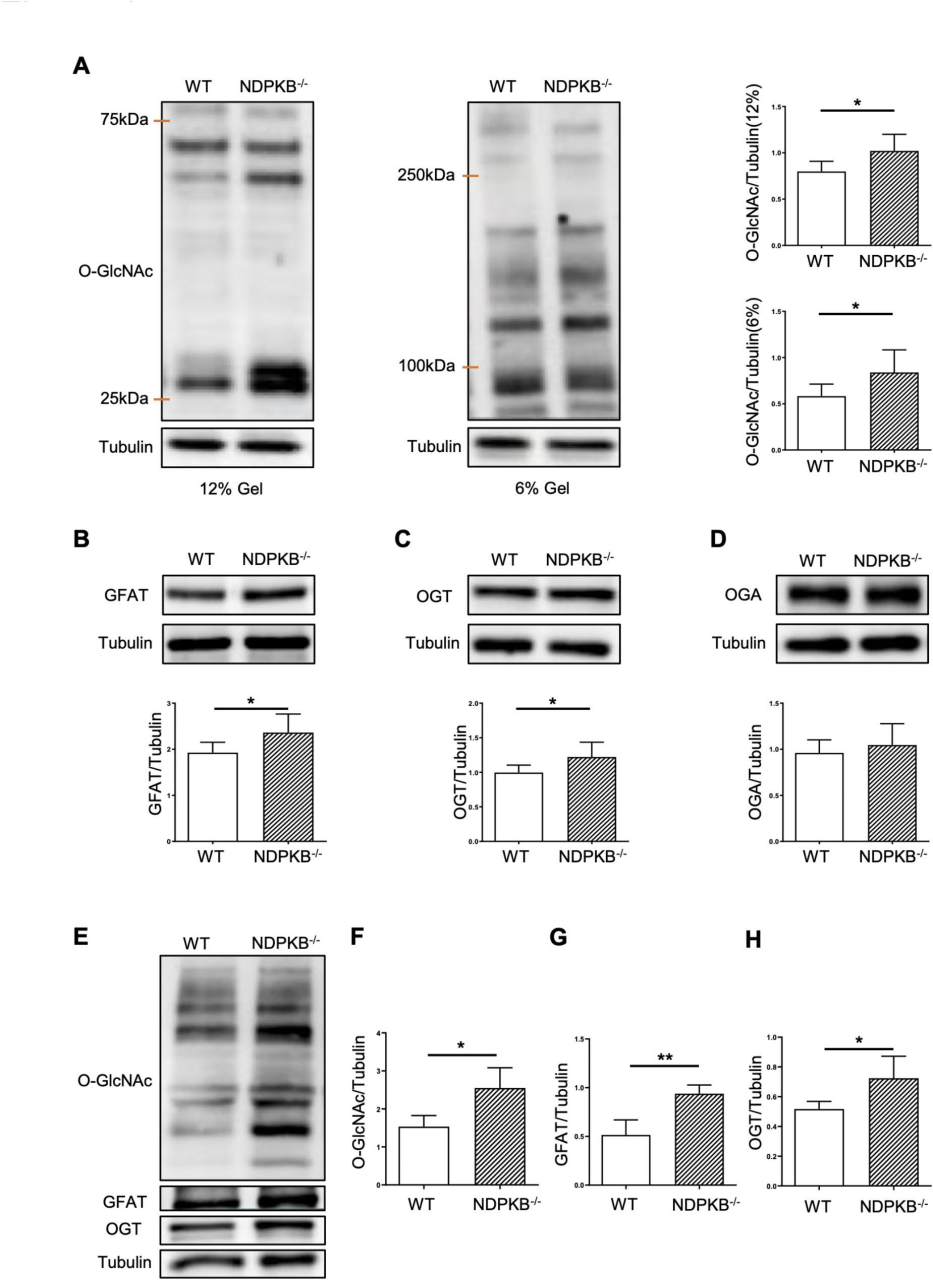
The hexosamine biosynthesis and downstream pathways are activated in NDPKB deficient hearts. Immunoblotting and statistical analysis of O-GlcNAc in 6% gel and 12% gel (**A**) with NDPKB^−/−^ LV proteins. Immunoblotting and statistical analysis of GFAT (**B**), OGT (**C**) and OGA (**D**) with NDPKB^−/−^ LV proteins. n = 6. Immunoblotting (**E**) and statistical analysis of O-GlcNAc (**F**), GFAT (**G**) and OGT (**H**) in NDPKB^−/−^ CECs. n = 4. *p < 0.05, **p < 0.01

Building on our previous findings, showing that deficient NDPKB in ECs leads to the activation of the HBP and vascular destruction in the retina [26, 27], we decided to estimate the levels of protein O-GlcNAcylation in LVs by immunoblotting. Using 6% and 12% SDS-PAGE for optimal separation of proteins, we observed significantly increased levels of O-GlcNAcylation in proteins ranging from 25–75 kDa and 90–250 kDa in NDPKB deficient LVs (Fig. 4A). Additionally, the expression of GFAT, a rate-limiting enzyme in the HBP, was significantly upregulated in LVs of NDPKB^−/−^ mice (Fig. 4B), indicating HBP activation in the heart. Moreover, we examined the downstream targets of the HBP activation, the OGT and OGA. The outcome of the immunoblotting experiments showed that the LV expression of OGT was significantly increased in NDPKB^−/−^ mice, whereas the LV expression of OGA was similar between these two groups (Fig. 4C and D), further supporting cardiac activation of the HBP upon NDPKB deficiency [27, 38].

Finally, we analyzed HBP-related proteins in CECs and CFs isolated from the NDPKB^−/−^ mice. Both O-GlcNAc and GFAT levels increased by approximately 60% in NDPKB^−/−^ CECs compared to their counterparts. The expression of OGT was significantly elevated by approximately 38% in NDPKB^−/−^ CECs, while OGA levels remained unchanged (data not shown), suggesting the activation of the endothelial HBP in the NDPKB^−/−^ mice. (Fig. 4E–H). However, the HBP and the subsequent O-GlcNac cycle remained unchanged in NDPKB^−/−^ CFs, indicating that CFs are insensitive to alterations in glucose metabolism in the context of HBP activation in NDPKB deficient hearts (Supplementary Fig. 4).

### Endothelial activation of the HBP leads to CM dysfunction

**Fig. 5:**
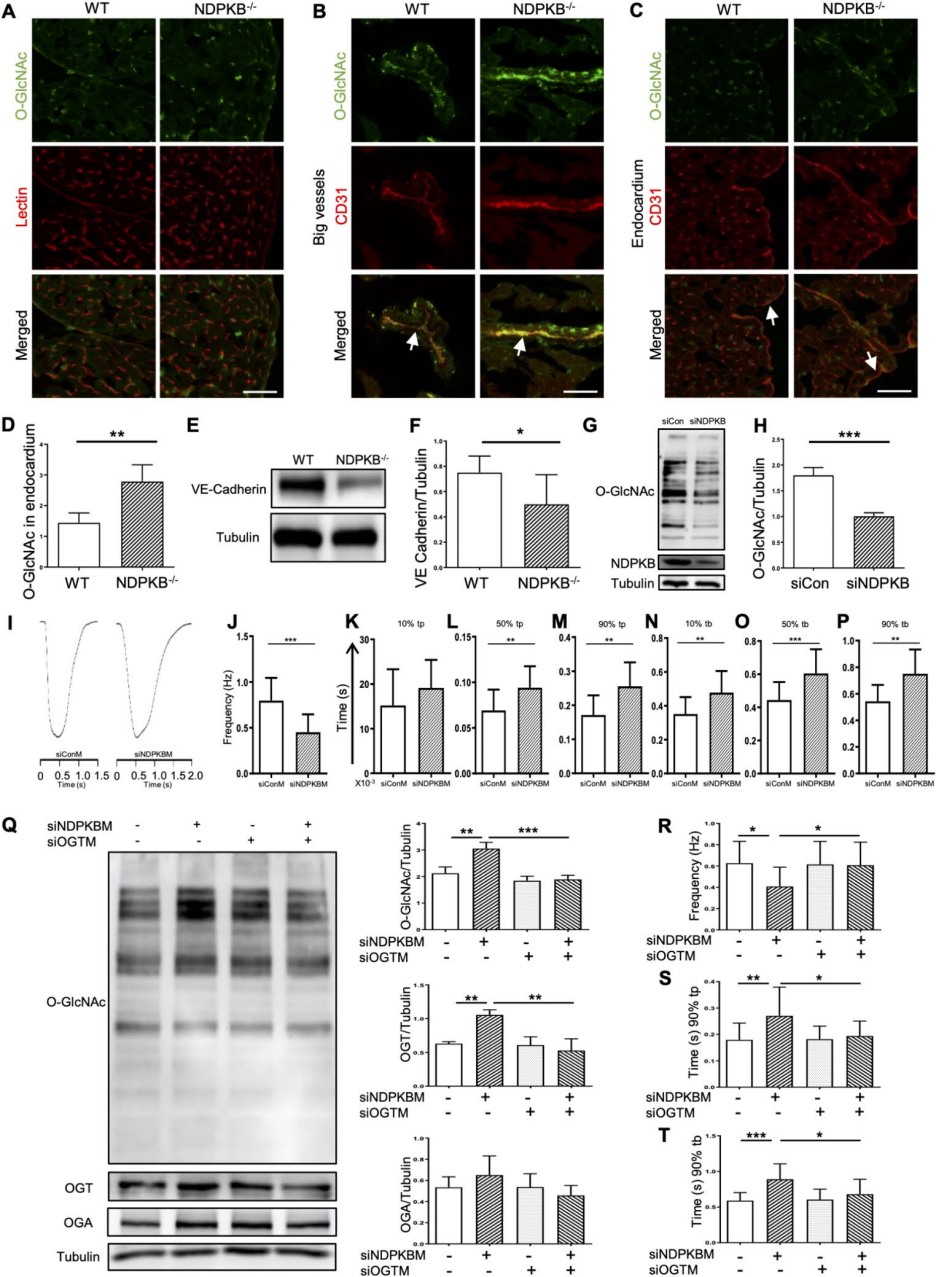
Endothelial activation of the HBP leads to CM dysfunction. Immunofluorescence staining of O-GlcNAc and lectin (a vascular marker) in NDPKB^−/−^ LVs (**A**). Green: O-GlcNAc, red: lectin. Immunofluorescence staining of O-GlcNAc and CD31 (endothelial marker) in big vessels of NDPKB^−/−^ LVs (**B**), Green: O-GlcNAc, Red: CD31, Arrows: big vessels. Immunofluorescence staining (**C**) and statistical analysis (**D**) of O-GlcNAc in endocardium of NDPKB^−/−^ LVs. Green: O-GlcNAc, Red: CD31. Arrows: interior surface of the heart. n = 5, repeated for three times. Scale bar = 50 µm. Immunoblotting (**E**) and statistical analysis (**F**) of VE-Cadherin in NDPKB^−/−^ LVs. n = 6. *p < 0.05. Immunoblotting (**G**) of NDPKB and O-GlcNac in NDPKB-depleted hiPSC-CMs, and statistical analysis (**H**) of protein O-GlcNAcylation. Representative traces of a complete contraction/relaxation cycle in single hiPSC-CM treated with conditioned medium of HUVECs, with or without NDPKB depletion (**I**). Characterization of CM contractility includes beat frequency (**J**), contraction time from baseline to 10% (**K**), 50% (**L**) and 90% (**M**) peak, and relaxation time from peak to 10% (**N**), 50% (**O**) and 90% (**P**) baseline. (**Q**) Immunoblotting and statistical analysis of O-GlcNAc, OGT and OGA in hiPSC-CMs treated with conditioned medium of HUVECs. n = 3. Statistical analysis of hiPSC-CM beat frequency (**R**), contraction time from baseline to 90% peak (**S**), and relaxation time from peak to 90% baseline (**T**). n = 15 for hiPSC-CM contraction/relaxation assessment, repeated in three individual hiPSC-CM lines. tp: to peak. tb: to baseline. siOGTM: conditioned siOGT-depleted endothelial medium. siNDPKBM: conditioned siNDPKB-depleted endothelial medium. siConM: conditioned endothelial control medium. *p < 0.05, **p < 0.01, ***p < 0.001

To further identify the sites of elevated protein O-GlcNAcylation overall in the LVs, we co-stained O-GlcNAc with lectin on the cardiac cryosections and found that O-GlcNAcylated proteins were predominantly localized in LV vessels (Fig. 5A and Supplementary Fig. 5). In addition, we assessed the capillary density in the myocardium but observed no significant differences, suggesting no obvious vascular morphological alternations in the NDPKB^−/−^ LVs (Supplementary Fig. 6). Using the EC marker CD31, it was noted that proteins in ECs were O-GlcNAcylated in large vessels such as arterioles (Fig. 5B) and in endocardium (Fig. 5C). Protein O-GlcNAcylation was also highly present in arteriolar smooth muscle cells recognized by the vascular morphology. The extent of protein O-GlcNAc modification in the ECs of NDPKB^−/−^ LVs was significantly greater than in controls in endocardium (Fig. 5C and D), indicating that the ECs in LVs might be the primary site for the activation of the HBP. We further assessed the expression of vascular endothelial cadherin (VE-cadherin), an adherens junction molecule in ECs, a critical marker for vascular damage in both glucose-dependent and independent contexts, in the LVs by immunoblotting [39]. The results showed significant downregulation of VE-cadherin in the NDPKB^−/−^ LVs (Fig. 5E and F), suggesting that vascular impairment may contribute to the HBP-induced cardiac dysfunction observed in NDPKB^−/−^ mice.

Modification of proteins at serine/threonine residues by O-GlcNAcylation has been proposed to potentially affect protein phosphorylation and modulate its function [40]. We initially estimated the overall protein O-GlcNAcylation in CMs under NDPKB deficient conditions using hiPSC-CMs. As shown in Fig. 5G, NDPKB was successfully depleted by 87% in hiPSC-CMs using the NDPKB specific siRNA. Surprisingly, unlike in ECs, O-GlcNAc modification of proteins was significantly decreased in NDPKB-depleted hiPSC-CMs. Nevertheless, NDPKB-depleted hiPSC-CMs exhibited noticeably slower beating rates compared with control cells, as depicted in the videos in supplementary Fig. 7, suggesting that CM dysfunction might not be a result of overall increased protein O-GlcNAcylation in CMs. Based on the previous finding showing endothelial damage under NDPKB deficient condition, we further evaluated the contribution of endothelial HBP activation in CM dysfunction. To finally explore whether endothelial activation of the HBP governs CM function in a paracrine manner, we measured the contraction and relaxation of single hiPSC-CM following treatment with conditioned medium of ECs with or without NDPKB depletion, denoted as siNDPKBM and siConM, respectively. As depicted in Fig. I and J, siNDPKBM markedly prolonged the beating period of hiPSC-CMs compared with siConM, showing a significant reduction more than 40% in spontaneous beating frequency. Delving into more details, siNDPKBM induced a significant extension of the contraction period of hiPSC-CMs at the time to 50% and 90% peak, but not to the early 10% peak (Fig. 5K–M). Simultaneously, a significant suppression of relaxation was observed throughout the entire relaxation period examined at the time to 10%, 50% and 90% baseline (Fig. 5N–P). The relaxation period seemed more sensitive than the contraction period in hiPSC-CMs incubated in siNDPKBM, as only the time to 10% baseline in the relaxation stage was elongated while the time to 10% peak in the contraction stage was not. These observations suggest that endothelial HBP may play a critical role in modulating the contractility of CMs, contributing to EC-CM interactions in maintaining normal cardiac function.

To further confirm that HBP activation in ECs contributes to cardiac dysfunction, we inhibited endothelial HBP activation using an OGT siRNA in NDPKB-depleted HUVECs, then subjected their supernatants to hiPSC-CMs. Protein O-GlcNAcylation increased around 67% by NDPKB deficiency and was reduced by approximately half under OGT and NDPKB double deficient conditions, demonstrating the loss of OGT suppressed HBP activation caused by NDPKB depletion (Supplementary Fig. 8A and 8B). Elevated fibronectin by NDPKB depletion in ECs was entirely normalized under OGT deficient conditions, suggesting an essential role of HBP activation in the accumulation of ECM (Supplementary Fig. 8C). The supernatants used for hiPSC-CMs treatment were denoted as siConM, siNDPKBM, siOGTM, and siNDPKBsiOGTM, respectively. In hiPSC-CMs treated with EC supernatants, siNDPKBM led to HBP activation in hiPSC-CMs, evidenced by elevation of O-GlcNAc and OGT, although OGA expression was not changed, showing the bipolar O-GlcNAc regulation in CMs. Furthermore, siOGTsiNDPKBM significantly inhibited the siNDPKBM-induced elevation of protein O-GlcNAcylation and OGT (Fig. 5Q). Our findings confirmed that HBP activation in hiPSC-CMs induced by endothelial siNDPKBM can be effectively inhibited via OGT depletion in ECs, highlighting the essential role of the endothelial HBP activation in modulation of HBP activation in CMs under NDPKB deficiency.

We further explored whether the inhibition of HBP activation via OGT depletion could mitigate the impaired contraction and relaxation properties of hiPSC-CMs induced by endothelial NDPKB depletion. SiOGTM significantly increased beating frequency which was reduced by siNDPKBM (Fig. 5R). As shown in Fig. 5S, the prolonged contraction phase of hiPSC-CMs at the time to 90% of the peak induced by siNDPKBM was rescued when hiPSC-CMs were treated with siOGTsiNDPKBM (Fig. 5S). Additionally, the significantly protracted relaxation observed in siNDPKBM-treated hiPSC-CMs at the time to 90% of baseline was significantly reversed by siOGTsiNDPKBM (Fig. 5T). The siOGTM treatment showed no difference from siConM in both contraction and relaxation. These results demonstrated that the disrupted CM kinetics caused by conditioned medium from NDPKB-depleted ECs can be prevented by inhibiting HBP activation in ECs. Collectively, these data suggested that endothelial HBP plays a crucial role in modulating CM contractility, underscoring its significance in EC-CM interactions and maintenance of normal cardiac function.

## Discussion

In this study, we found that activated HBP with enhanced GFAT expression, subsequent promotion of protein O-GlcNAcylation and the O-GlcNAc cycle with elevated OGT expression were evident in NDPKB deficient LVs. Protein O-GlcNAcylation was detected mainly in cardiac ECs. NDPKB^−/−^ mice developed cardiac hypertrophy showing an increased heart weight and enlarged LVPW, along with diastolic dysfunction. Additionally, phosphorylation of PLN and expression levels of SERCA2 in the LVs were decreased in NDPKB^−/−^ mice. Accumulation of ECM proteins and increased expression of TGF-β were detected in NDPKB deficient LVs as well. A bipolar O-GlcNAc regulation by ECs and CM themselves was identified in the CMs. Conditioned medium from NDPKB-depleted ECs induced a decrease in CM beating frequency and impaired contraction, especially relaxation kinetics, which were attenuated by the inhibition of endothelial HBP activation. These data indicated a correlation between endothelial HBP activation and cardiac dysfunction.

An essential finding in this study is the cardiac dysfunction caused by HBP activation in NDPKB deficient mice assessed by echocardiogram and applications of molecular biology techniques. NDPKB deficiency induces diastolic dysfunction, showing significantly decreased E, E’, and E/A ratios. Diastolic dysfunction manifests in conditions such as hypertension and DC, leading to alterations in LV structure and function, particularly in the context of DC, where it is often considered a defining feature [41, 42]. Basu et al. reported that 6-month-old Akita mice exhibited diastolic dysfunction while maintaining normal systolic function, even in the absence of cardiac hypertrophy and fibrosis [43]. In contrast, Jaffe et al. found that STZ-induced diabetic rats showed diastolic dysfunction along with compromised LV systolic function [44]. The NDPKB^−/−^ mice were glucose intolerant, yet insulin secretion and ITT remained unaltered, suggesting an early stage of prediabetes in which insulin sensitivity may still be preserved during the ITT, even though glucose metabolism is impaired during the GTT. More detailed studies are still needed to characterize the mice. Nevertheless, the NDPKB^−/−^ mice displayed cardiac characteristics similar to those observed in pre-diabetic and diabetic cardiomyopathy. Our findings agree with another study that demonstrated cardiac dysfunction and ECM accumulation already manifests in the pre-diabetic stage [45]. Phosphorylation of PLN is essential in cardiac Ca^2+^ handling and excitation–contraction coupling via SERCA2. PLN phosphorylation suppresses its inhibitory effect on SERCA2, thereby accelerating Ca^2+^ reuptake during diastole [46, 47]. Yokoe et al., found that PLN is modified by O-GlcNAc, which in turn suppresses phosphorylation of PLN by PKA. Dysregulation of intracellular Ca^2+^ in rat CMs under elevated protein O-GlcNAcylation caused by pharmacological intervention or hyperglycemia implicates its role in the deterioration of cardiac function [21]. Decreased phosphorylation of PLN was detected in NDPKB^−/−^ LVs, which might potentially enhance the inhibitory effect of SERCA2. Moreover, the observed changes in PLN and SERCA2 expression levels and thus the increase in PLN to SERCA2 ratios may additionally add to the impaired Ca^2+^ cycling and consequently altered contraction and relaxation kinetics in NDPKB deficient CMs. It has been reported that NDPKB is involved in the production of cellular cAMP in CMs, while NTP homeostasis including ATP remained unaffected under NDPKB deficient conditions [27, 48]. This study supports the findings demonstrated by our previous reports about essential role of NDPKB in the regulation of cAMP levels [48, 49], indicating that cardiac dysfunction might be mediated by multiple manners rather than only regulation in cAMP signaling or protein O-GlcNAcylation in CMs. A link between cAMP regulation and HBP activation may exist, and the local and specific O-GlcNAcylation of certain proteins such as PLN cannot be excluded in NDPKB deficient CMs. Further studies are required to clarify the underlying mechanisms.

In DC, diastolic dysfunction is closely associated with cardiac hypertrophy as well [42]. Studies on DC have demonstrated increased diastolic LVPW thickness in patients with diabetes [50, 51] and also in rodent models [52]. A similar increase in diastolic LVPW was observed in NDPKB^−/−^ mice, indicating the initiation of cardiac hypertrophy. Furthermore, like cardiac hypertrophy observed in rodent models of DC, increased heart to body weight ratio, heart to tibia length ratio, enlarged CM size and LV mass were substantiated in NDPKB^−/−^ mouse in this study. In our study, the increase in CM size was observed in the subendocardial layer of the myocardium, but not in the subepicardial layer, which may reflect regional differences in local myocardial structure, metabolism, and functional activities as previously reported [53–55]. In this study, cardiac hypertrophy in pre-diabetic NDPKB^−/−^ mice might be associated with cardiac dysfunction and upregulation of protein O-GlcNAcylation. These findings further corroborate the research reported by Ha et al., which demonstrated that sustained elevation of LV GlcNAc levels contributes to cardiac hypertrophy and diastolic dysfunction, underscoring the critical role of HBP activation in the development of diastolic dysfunction [56]. Further experiments using hiPSC-CMs treated with conditioned medium from NDPKB- and OGT-depleted ECs showed an HBP-dependent improvement of CM beating frequency and contraction/relaxation kinetics, supporting the findings in vivo.

We additionally observed an accumulation of fibronectin and CTGF in the LVs of NDPKB deficient mice. Studies in diabetic patients and animal models of DC showed that increased expression of ECM proteins such as fibronectin and collagen is associated with cardiac fibrosis [57–59]. Both CFs and CECs contributed to ECM accumulation in the NDPKB deficient conditions. However, key HBP-related markers remained unchanged in CFs, suggesting that the expression of fibronectin in CFs might be regulated through other signaling pathways rather than HBP activation in NDPKB deficient hearts. Notably, the ECs are the primary site for HBP activation, as evidenced by in vitro and in vivo experiments in the study. Despite the widespread distribution of ECs in the LVs, whether structural and functional heterogeneity of ECs is involved in the local fibrotic response in the NDPKB^−/−^ LVs requires further investigation. We observed higher fibronectin expression in the subepicardial layer compared to the subendocardial layer in control mouse hearts. Accumulation of fibronectin has been associated with increased mechanical stress and tissue injury [60, 61]. Beyhoff et al. demonstrated that subendocardial and subepicardial regions exhibit different susceptibility to blood pressure [62]. In our study, hypertrophy in NDPKB deficient LVs, as shown in Fig. 1, originated in the subendocardial region. The observed differences in fibronectin expression and the initiation of hypertrophy in NDPKB deficient LVs may reflect variations in mechanical and metabolic activities across the left ventricular wall. Recent studies suggest that cardiac fibrosis can be attenuated, and thus cardiac function is improved [63]. The results on inhibited fibronectin expression by OGT suppression in NDPKB-depleted ECs support this report. The NDPKB deficiency-induced increase in relevant markers of ECM accumulation seems to be another critical factor in promoting the cardiac dysfunction observed in this model. We observed changes in TGF-β, a profibrotic cytokine and the most important inducer of fibrosis, in NDPKB deficient LVs. Global TGF-β activity is increased in various cardiac fibrosis models and in human hearts with fibrotic alterations, where the fibrotic response and ventricular remodeling occur [64–67]. TGF-β deficient mice showed decreased myocardial fibrosis during ventricular remodeling with aging [68]. Conversely, overexpression of TGF-β1 in mice promotes cardiac fibrosis in LVs [69]. Our data on upregulated TGF-β in NDPKB^−/−^ LVs align with other studies verifying its essential role in cardiac fibrosis and suggesting the accumulation of ECM proteins is correlated to increased TGF-β levels in NDPKB^−/−^ LVs.

As shown above, the NDPKB^−/−^ hearts exhibited abnormal glucose tolerance, as well as cardiac functional and structural abnormalities, imitating the human pre-diabetic and diabetic cardiomyopathy. This study provides a promising animal model mimicking the diseases with multiple, significant pathological alterations seen in patients. Previous studies from our group have demonstrated the correlation between NDPKB and intracellular glucose metabolism, particularly the activation of HBP in ECs and the retina [27]. In this study, we found that NDPKB deficient hearts exhibited upregulation of GFAT, excessive protein O-GlcNAcylation, and elevated OGT expression in LVs. These findings are consistent with reports showing cardiac HBP activation and subsequent adaptive regulation in the O-GlcNAc cycle in diabetic animals and humans [16, 22]. Deficiency of NDPKB in mice boosts HBP flow via the elevation of GFAT and OGT expression, resulting in enhanced protein O-GlcNAc modification in the NDPKB deficient heart. To the best of our knowledge, we are the first to show that the vasculature is the predominant O-GlcNAcylation target in the myocardium of LVs. The activation of HBP was identified in cardiac ECs in NDPKB deficient mice. A study by Federici et al. demonstrated that the activation of the HBP under hyperglycemia increases O-GlcNAcylation but simultaneously decreases phosphorylation of proteins correlated to insulin signaling in diabetic LVs in vivo and in ECs in vitro, leading to vascular damage of DC [70]. The downregulation of VE-cadherin in pathological conditions directly facilitates the loss of endothelial intercellular adhesions, and ECs lose their typical phenotypes and functions [71]. Decreased expression of VE-cadherin in the LVs of NDPKB deficient mouse signifies vascular damage in cardiac dysfunction. A decline in cardiac VE-cadherin expression without alternations in vascular density in LVs further suggests an early-stage vascular damage in NDPKB^−/−^ mice, mapping its cardiac feature in pre-diabetes. Additionally, our observation on overall increased protein O-GlcNAcylation in NDPKB^−/−^ LVs might cause dysregulation of intracellular Ca^2+^ signaling, resulting in cardiac dysfunction with cardiac hypertrophy and abnormal ECM deposition. Juni et al. demonstrated that EC-conditioned medium is able to enhance CM contractility compared with controls [72]. The data on the function of hiPSC-CMs treated with conditioned medium from NDPKB/OGT-depleted ECs support these findings. Importantly, our study provides evidence that vascular damage associated with the activation of the endothelial HBP may promote cardiac dysfunction in the LVs. The study suggests the secretome of NDPKB deficient ECs might regulate CM function. ECs release a range of paracrine factors that influence key processes such as growth/survival, glucose metabolism, inflammation, hypertrophy, and fibrosis, thereby modulating CM function. A comprehensive study investigating the mediation of these factors through HBP activation, using proteomic analysis and a conditional endothelial-specific NDPKB deficient mouse model, will offer valuable insights into the underlying mechanisms.

Notably, different cardiac cells exhibit varying HBP responses under NDPKB deficiency. Interestingly, a bipolar O-GlcNAc regulation was identified in CMs, involving interactions with surrounding cardiac cells and intrinsic changes within the CMs themselves. While NDPKB depletion reduced O-GlcNAcylation in hiPSC-CMs, the secretome of NDPKB-depleted ECs elevated O-GlcNAcylation in hiPSC-CMs, despite CM dysfunction. This contrasts with the regulatory behavior seen in ECs, where elevated O-GlcNAcylation was evident. This discrepancy may be attributed to differences in metabolic regulation between ECs and CMs. ECs are critical for maintaining proper cellular signaling, inflammatory responses, and vascular integrity [70]. ECs are more dependent on glycolysis and HBP, while fatty acid oxidation is the predominant source of energy in healthy CMs [73]. These metabolic differences may contribute to the observed variation in O-GlcNAcylation expression between these cell types.

**Fig. 6:**
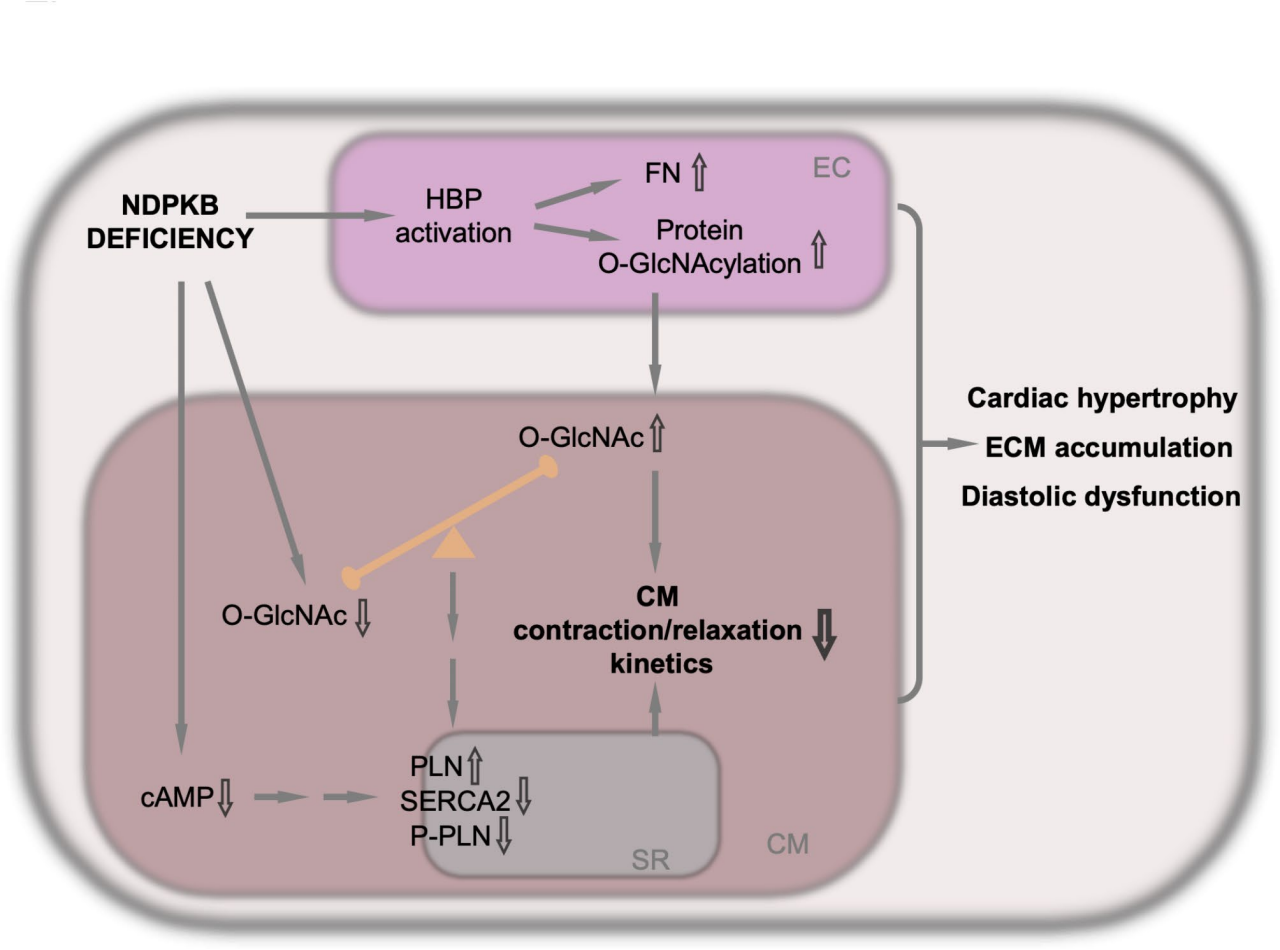
Illustration of endothelial activation of the HBP and cardiac dysfunction in pre-diabetic NDPKB deficient mice. Under NDPKB deficiency, protein O-GlcNAcylation increases in the heart, predominantly in ECs, but is bipolar regulated in CMs. O-GlcNAc levels decrease in NDPKB-depleted CMs, whereas secretome from the NDPKB-depleted ECs leads to an increase in O-GlcNAcylation in CMs, contributing to CM dysfunction. Additionally, NDPKB deficiency in CMs induces a decline in cAMP levels [48], which might regulate the expression of PLN and phosphorylation of PLN along with downregulation of SERCA2 in LVs, thereby further impairing contraction and relaxation kinetics of CMs. The alterations in ECs as well as CMs contribute to cardiac hypertrophy, ECM accumulation and diastolic dysfunction under NDPKB deficient conditions. HBP: hexosamine biosynthesis pathway; NDPKB: nucleoside diphosphate kinase B; EC: endothelial cell; CM: cardiomyocyte; PLN: phospholamban; cAMP: cyclic adenosine monophosphate; SERCA2: sarcoplasmic/endoplasmic reticulum Ca2 + -ATPase 2; ECM: extracellular matrix; FN: fibronectin

In summary, cardiac hypertrophy and accumulation of ECM proteins in NDPKB deficient LVs are likely due to HBP activation in the vasculature, particularly in ECs, leading to CM dysfunction (Fig. 6). CM dysfunction could be promoted by dual manners. Both, alterations in cAMP mediated signaling and dysregulation of protein O-GlcNAcylation in ECs and CMs might participate in CM dysfunction under NDPKB deficient condition [48]. The findings emphasize the possible role of endothelial HBP in maintaining cardiovascular homeostasis, potentially leading to new therapeutic targets in pre-diabetic and diabetic cardiomyopathy. Nevertheless, further investigations are needed to explore the link between NDPKB deficiency and metabolic abnormalities, in particular the activation of the endothelial HBP, to provide solid evidence for the inhibition of endothelial HBP activation in pre-diabetic and diabetic cardiomyopathy and NDPKB deficient conditions.

## Electronic supplementary material

Below is the link to the electronic supplementary material.

Supplementary Material 1. Fig. 1. NDPKB^-/-^ mice are glucose intolerant, but have normal insulin secretion. Statistical analysis of GTT (A) and blood insulin levels after GTT (B) in NDPKB-/- mice. (C) Statistical analysis of ITT in 8-month NDPKB-/- mice. n=7-8. Statistical analysis of the insulin secretion in islets isolated from NDPKB^-/-^ mice (D). n=23-30. Statistical analysis of the glucagon secretion in islets isolated from NDPKB^-/-^ mice (E). n=15-20. *p<0.05. Fig. 2. Fibronectin is expressed in vasculature. Immunofluorescence staining of fibronectin and lectin (a vascular marker) in the mouse LVs. Green: lectin, red: fibronectin. n=3. Scale bar=20µm. Fig. 3. Accumulation of collagen is evident in the LVs of NDPKB^-/-^ mice. Sirius-red staining of whole heart (A) and statistical analysis in NDPKB^-/-^ and WT LVs (B). n=5. ***p<0.001. Scale bar=500µm. Fig. 4. HBP-related proteins remain unchanged in CFs isolated from NDPKB¬ /- mice. Immunoblotting (A) and statistical analysis of O-GlcNAc (B), GFAT (C), OGT (D), and OGA (E) in CFs isolated from NDPKB^-/-^ mice. n=4. Fig 5. Predominant Localization of O-GlcNAc is in vessels of the LVs. Video shows co-localization of O-GlcNAc and lectin in LV. Green: O-GlcNAc, red: lectin. Scale bar=10 µm. Fig. 6. Capillary density shows no change in NDPKB^-/-^ LVs. A: Immunofluorescence staining of lectin (a vascular marker) in NDPKB^-/-^ LVs. Statistical analysis of capillary density in the subendocardial (B) and subepicardial layer (C). n=3. Scale bar=20µm. Fig. 7. Beating rates of CMs decrease dramatically in NDPKB-depleted hiPSC-CMs. Videos show beating rates of CMs transfected with NDPKB siRNA (siNDPKB) or control siRNA (siCon). Fig. 8. The NDPKB deficiency-induced HBP activation and fibronectin upregulation is inhibited by OGT depletion in HUVECs. Immunoblotting (A) and statistical analysis of O-GlcNAc (B) and fibronectin (C) in NDPKB-depleted, OGT-depleted, NDPKB/OGT double-depleted and control HUVECs. n = 4. *p < 0.05, **p<0.01, ***p < 0.001.

## Data Availability

The datasets used and/or analyzed during the current study are available from the corresponding author on reasonable request.

## Abbreviations

CM: Cardiomyocyte
DC: Diabetic cardiomyopathy
ECM: Extracellular matrix
ECs: Endothelial cells
CEC: Cardiac endothelial cell
CF: Cardiac fibroblast
GFAT: Glucose-6-phosphate-Glutamine: fructose-6-phosphate amidotransferase
GTT: Glucose tolerance test
HBP: Hexosamine biosynthesis pathway
HbA1c: Glycated hemoglobin
HUVECs: Human umbilical cord vein endothelial cells
HiPSC: Human Induced Pluripotent Stem Cell
hiPSC-CM: HiPSC-derived cardiomyocyte
ITT: Insulin tolerance test
LVs: Left ventricles
LVAW; systole: Systolic LV anterior wall
LVAW; diastole: Diastolic LV anterior wall
LVPW; systole: Systolic LV posterior wall
LVPW; diastole: Diastolic LV posterior wall
MV: Mitral valve
NDPKs: Nucleoside diphosphate kinases
NDPKB: Nucleoside diphosphate kinase B
NDPKB^−^^/^^−^: NDPKB deficient
O-GlcNAc: O-linked beta-N-acetylglucosamine
OGT: O-GlcNAc transferase
OGA: O-GlcNAcase
PLN: Phospholamban
PW-Doppler: Pulse wave-Doppler
siConM: SiRNA control conditioned medium
siNDPKBM: SiNDPKB-depleted conditioned medium
SR: Sarcoplasmic reticulum
STZ: Streptozotocin
SERCA2: Sarcoplasmic/endoplasmic reticulum Ca^2+^-ATPase 2
