# Correction: Deficiency in nucleoside diphosphate kinase B leads to endothelial activation of the hexosamine biosynthesis pathway and cardiac dysfunction

**DOI:** 10.1186/s12933-025-02759-9

**Published:** 2025-05-13

**Authors:** Feng Shao, Johanna Wieland, Yixin Wang, Merve Keles, Zenghui Meng, Santosh Lomada, Miao Qin, Veronika Leiss, Abel Martin-Garrido, Manuela Fuhrmann, Yi Qiu, Felix A. Trogisch, Christiane Vettel, Joerg Heineke, Yuxi Feng

**Affiliations:** 1 Experimental Pharmacology Mannheim, European Center for Angioscience (ECAS), Medical Faculty Mannheim, Heidelberg University, Ludolf-Krehl-Str. 13-17, 68167 Mannheim, Germany; 2 Department of Cardiovascular Physiology, European Center for Angioscience (ECAS), Medical Faculty Mannheim, Heidelberg University, 68167 Mannheim, Germany; 3 DZHK (German Center of Cardiovascular Research), Partner Site Heidelberg/Mannheim, Mannheim, Germany; 4 First Department of Medicine, Faculty of Medicine, University Medical Centre Mannheim (UMM), University of Heidelberg, 68167 Mannheim, Germany; 5 Department of Pharmacology, Experimental Therapy and Toxicology, University of Tübingen, 72074 Tübingen, Germany

**Correction to: Cardiovascular Diabetology (2025) 24:84** 10.1186/s12933-025-02633-8

In the original publication of this article [1], the author’s name “**Felix A. Trogisch**” was incorrectly written as “Trogisch Felix”.

The original article has been corrected.
